# Behavioral and Emotional Dysfunction in Parkinson's Disease

**DOI:** 10.1155/2019/1749525

**Published:** 2019-06-18

**Authors:** Matteo Bologna, Aristide Merola, Lucia Ricciardi

**Affiliations:** ^1^Department of Human Neurosciences, Sapienza University of Rome, Rome, Italy; ^2^IRCCS Neuromed, Pozzilli (IS), Italy; ^3^Department of Neurology, Gardner Family Center for Parkinson's Disease and Movement Disorders, University of Cincinnati, Cincinnati, OH, USA; ^4^Neurosciences Research Centre, Molecular and Clinical Sciences Research Institute, St George's University of London, London, UK

Alongside the major interest for Parkinson's disease (PD) motor symptoms, in recent years, there has been increasing attention to PD-associated nonmotor symptoms. Behavioral and emotional dysfunctions have been identified as major determinants of health-related quality-of-life impairment, frequently affecting PD patients since the very first stages of the disease or even before the onset of classical motor symptoms [1, 2, 3]. Despite their major impact on the quality of life, behavioral and emotional disorders may be underrecognized and untreated in PD.

Although the exact pathogenesis of behavioral and emotional disorders associated with PD remains to be clarified, several mechanisms have been identified. Because of the complex role of basal ganglia in processing a wide range of motor and nonmotor information [4, 5], dysfunction in these structures is thought to play a key role in generating behavioral and emotional disorders in PD. Other mechanisms, however, include dopaminergic and nondopaminergic dysfunctions of several pathways at the subcortical and cortical levels, including the limbic system [6, 7].

It is worth considering that PD affects over 6 million people worldwide and that this number is projected to double by 2040 [8]. The need for expanding the knowledge on behavioral and emotional dysfunctions represents, therefore, a key priority for clinicians and researchers and is crucial to the effective treatment of these disturbances.

This special issue was specifically designed to focus on three main critical areas, namely, (a) evidence of PD-associated behavioral and emotional dysfunctions, (b) neural correlates and pathophysiological aspects of behavioral and emotional dysfunction associated with PD, and (c) clinimetric assessment and screening instruments for PD-associated behavioral and emotional dysfunctions.

From the initial analysis of thirteen papers submitted by international researchers, seven were identified as the most relevant and peer-reviewed by experts in the field. The following is a short summary of the major findings of each of these papers.

Imaging genetics is a novel-integrated research method which combines different techniques in the attempt to disentangle the relationship between genetic factors and brain functions or structures. By using this experimental approach, Y. Zhi et al. further investigated the pathophysiological basis of depression in patients. The authors employed resting-state functional magnetic resonance imaging and tested the dopamine receptor D3 gene polymorphism. They found that the D3 gene Ser9Gly polymorphism was associated with more severe anhedonia in PD patients. They discussed the possible role of the frontal areas, namely, the right inferior occipital gyrus, lingual gyrus, and fusiform gyrus, in generating depressive symptoms in PD. The association between genetic variants imaging phenotypes and different clinical manifestations of nonmotor symptoms should be further explored in future studies.

Hallucinations are common disturbances in patients with PD. Previous studies have mainly investigated the neural correlates of well-formed visual hallucinations in PD patients with advanced disease. By contrast, less is known about patients in the early stages of the disease and about minor hallucinations. C. M. Sawczak et al. investigated the contribution of the attention networks in generating minor hallucinations in PD. In their neuroimaging study, the authors demonstrated a thicker cortex in both the dorsal and ventral attention networks. It is noteworthy to remark, however, that prior work showed the opposite result, i.e., grey matter reductions in PD. Therefore, the available data so far indicate a variable pattern of cortical thickness alterations associated with hallucinations in PD. Further investigations and longitudinal studies are warranted to better define the neural correlates of PD psychosis.

Even though the decline in motor performance is the most prominent feature of PD progression, the relationship between movement abnormalities and nonmotor symptoms, including a deficit in motivation, is far from being understood. In their original study, M. Kojovic et al. tested the effect of monetary incentive on movement speed in PD patients treated with STN-DBS and dopaminergic medications. The results indicate that motivational modulation of movement speed may be enhanced as a direct consequence of DBS rather than dopaminergic treatment. The results may be relevant for future and innovative rehabilitation programs.

In their viewpoint paper entitled “Motor and nonmotor symptoms of PD; antagonistic pleiotropy phenomena derived from *α*-synuclein evolvability?,” Y. Takamatsu et al. discuss nonmotor symptoms, including depression and anxiety, in PD and other synucleinopathies in an evolutionary perspective. The authors also discuss possible implications related to novel therapeutic strategies.

Mounting shreds of evidence suggest that people with PD perform poorly on tests assessing the ability to infer the beliefs, desires, and intentions of others. All these functions fall under the umbrella term of “Theory of Mind” (ToM). In their original paper, J. A. Foley et al. reveal that apparent impairment observed on ToM tests in PD patients is explained by altered executive functions. Among these, the authors report deficits in inhibition, likely due to neuropathological, metabolism, or connectivity changes of the frontal lobe in PD. The results highlight the importance of accurate neuropsychological testing in PD patients.

Proper assessment of behavioral and emotional dysfunctions in PD is a challenging issue, in both clinical and research contexts. For these purposes, various scales exist; however, their reliability and validity are not always tested in specific populations where contextual, social, and environmental factors may significantly influence the outcome measure. Thus, psychometric properties of the clinical scales have been often studied in different pathologies and in different settings. P. Massai et al. investigated the internal consistency, test-retest reliability, and construct and discriminant validity of the Geriatric Depression Scale (GDS) in Italian people with PD. Their results demonstrate that GDS is reliable and valid in Italian patients to quantify depression in PD.

With an estimated prevalence of ∼3–42%, impulse control behaviours (ICB) and disorders (ICD) represent one of the most significant nonmotor health problems in patients with PD. Questionnaire for Impulsive-Compulsive Disorders in PD (QUIP) is the most commonly used screening instrument for ICD in PD patients. E. Maréchal et al. translated the QUIP into Flemish to develop a useful screening instrument for ICD in the Belgian population. They used a translation-backtranslation method. The results of this pilot study suggest that the validity of the questionnaire is similar to the original version. The authors emphasize the role of a proper screening instrument for the detection of ICB and ICD in the people with PD.

We believe that this special issue will contribute to raise interest in several aspects related to behavioral and emotional dysfunction in PD. We aimed to highlight original research findings from a broad perspective. We hope that this special issue might inspire future and novel research approaches to tackle some of the unsolved, and still controversial, issues highlighted in the various studies.

In conclusion, we would like to thank all the authors, the reviewers, and the editorial board members for their effort in constructing this special issue on behavioral and emotional dysfunction in PD.
